# Writing Practices Associated With Electronic Progress Notes and the Preferences of Those Who Read Them: Descriptive Study

**DOI:** 10.2196/30165

**Published:** 2021-10-06

**Authors:** Thomas H Payne, Carolyn Keller, Pallavi Arora, Allison Brusati, Jesse Levin, Monica Salgaonkar, Xi Li, Jennifer Zech, A Fischer Lees

**Affiliations:** 1 Department of Medicine University of Washington School of Medicine Seattle, WA United States; 2 University of Southern California Los Angeles, CA United States; 3 Columbia University New York, NY United States

**Keywords:** electronic documentation, electronic health records, hospital progress notes, copy-paste, EHR, patient records, workflow, human factors, clinical communication, physician communication, hospital

## Abstract

**Background:**

Hospital progress notes can serve as an important communication tool. However, they are criticized for their length, preserved content, and for the time physicians spend writing them.

**Objective:**

We aimed to describe hospital progress note content, writing and reading practices, and the preferences of those who create and read them prior to the implementation of a new electronic health record system.

**Methods:**

Using a sample of hospital progress notes from 1000 randomly selected admissions, we measured note length, similarity of content in successive daily notes for the same patient, the time notes were signed and read, and who read them. We conducted focus group sessions with note writers, readers, and clinical leaders to understand their preferences.

**Results:**

We analyzed 4938 inpatient progress notes from 418 authors. The average length was 886 words, and most were in the Assessment & Plan note section. A total of 29% of notes (n=1432) were signed after 4 PM. Notes signed later in the day were read less often. Notes were highly similar from one day to the next, and 26% (23/88) had clinical risk associated with the preserved content. Note content of the highest value varied according to the reader’s professional role.

**Conclusions:**

Progress note length varied widely. Notes were often signed late in the day when they were read less often and were highly similar to the note from the previous day. Measuring note length, signing time, when and by whom notes are read, and the amount and safety of preserved content will be useful metrics for measuring how the new electronic health record system is used, and can aid improvements.

## Introduction

Inpatient progress notes can serve as an important communication tool across the physician team, nurses, therapists, consultants, and the patient. Because safety problems that occur in hospitals can be traced to communication lapses [1], progress notes are vital to achieve safer care. However, some feel that few read progress notes, and that they therefore no longer serve the purpose of communication and are now primarily billing documents [2]. Many physicians feel they spend too much time writing progress notes [3,4] and employ methods to shorten writing time. These include copy-paste [5-8] and extensive templating with “note bloat,” which can introduce error [9-11], harm patients [12], and make it difficult for note readers to separate current from outdated content [13].

We have used our current electronic health record (EHR) system at the University of Washington (UW) Medical Center and the Harborview Medical Center for 17 years with little change in the format of the inpatient progress note used in the Medicine service. In preparation for the transition to a new inpatient EHR system, we conducted this analysis of progress notes on inpatient medical services to take stock of current practices. The purpose of this study was to describe current hospital progress note writing and reading practices, as well as the preferences of those who create progress notes and those who read them.

## Methods

### Overview

This work retrieved its data from the inpatient general medicine services at the UW Medical Center and the Harborview Medical Center, which are major teaching hospitals of the UW with approximately 35,000 combined admissions annually. The EHR system was installed in 2003, and the transition from paper to electronic notes occurred in 2006 using Cerner Millennium (Cerner Corp). Nearly all progress notes pertaining to these inpatient services are typed using the Clinical Notes Editor, based on templates that automatically import patient-specific data such as medication lists, vital signs, and laboratory results [14]. Daily progress notes are required by hospital bylaws and are mostly written by residents (usually interns) and attending hospitalists.

### Selection of Notes

We randomly selected 1000 patient admissions to the general Medicine service of UW Medical Center and the Harborview Medical Center between July 1, 2016, and June 30, 2017. The Medicine services at both facilities share the same progress note template. We excluded patients admitted to subspecialty services with unique progress note templates (oncology, cardiology, and geriatrics). For each admission, we extracted the data for all daily progress notes as shown in Textbox 1 from the analytical data repository (Enterprise Data Warehouse, Caradigm), which contains a subset of EHR data extracted for research. Progress notes were identified by the title “Medicine - Inpt Record,” the note type used within our system for Medicine service daily progress notes. Given that this study was focused on practices around the use of progress notes by those who create and those who read them, we excluded all notes other than progress notes (admission notes, procedure notes, consult notes, interim summaries, discharge summaries, and other notes).

**Metadata obtained for each progress note.**

Full text of the note and other data listed were gathered for each note. Authenticators are supervising physicians whose note cosignature finalizes the note.

**Metadata obtained:**

- Note date and time
- Note title
- Note authors and authenticators
- Note identifier
- Note text
- Note action log (provided by the electronic health record system), which includes the following:
- Action (eg, perform, transcribe, modify, sign and CC/review, verify)
- Performed by (name)
- Performed date and time
- Action status
- Comment
- Proxy personnel
- Requested by (name)

### Note Analysis

We deidentified each note using published methods [15] and stored them securely. Using Python scripts written for this project, we determined the total number of words in each note and in each note section (Identification/Chief Concern, Interim History, etc) (Textbox 2). We also determined when notes were signed; before progress notes are signed, they cannot be viewed except by the author.

**Progress note sections.**

Sections are automatically created using the template used to create Medicine progress notes.

HOSPITAL DAY

IDENTIFICATION/CHIEF CONCERN

INTERVAL HISTORY

INPATIENT PROBLEM LIST

ALLERGIES

SCHEDULED MEDICATIONS

PRN MEDICATIONS

PHYSICAL EXAM

LABS

- Micro

IMAGING

ASSESSMENT & PLAN

- Fluids/electrolytes/nutrition
- Prophylaxis
- Tubes/lines
- Disposition
- Code status
- Contacts

ATTENDING STATEMENT

- Additional diagnoses

### Copy-Paste Analysis

We identified the sequence of daily notes written for each patient during their hospital stay and then determined the percentage of text within each and in the note as a whole that overlapped with the note written on that same patient the previous day using natural language processing methods [16,17].

To determine the clinical implications of copy-paste, we used methods described by Hammond et al [18] to highlight shared content across progress notes from one day compared to the previous day for the same patient. We then used the same 6-point scale as in that paper to rank the clinical importance of copy-paste. Since this required time-consuming manual review by clinicians, we performed this for a subset of notes.

### Measuring Note Readership

Note reading practices were analyzed by extracting a note-viewing record using auditing software (P2Sentinel, Cerner Corporation). Each time a note was viewed, the username, user role (resident physician, attending physician, registered nurse, etc), and timestamp were recorded in the auditing database. To determine views within the same hospital day—which have unique potential to communicate the patient’s current clinical state and today’s plan—we assessed note views within 12 hours after the note was signed. Usernames were used to identify members of the patient’s primary team. Views by physicians on the patient’s primary team were assumed to be related to the note writing process, and were excluded from the note-reading analysis. Statistical testing was performed using Stata/IC 13 (StataCorp LLC).

### Focus Groups

To understand different perspectives on the current use of progress notes, we conducted 3 sets of focus groups: *note authors* (Medicine interns and hospitalists), *note consumers* (nurses, therapists, and consultants who view Medicine progress notes that others had written), and *leaders* (hospital service leaders who form documentation policies and standards). Each focus group was led by a coinvestigator who followed a script and showed PowerPoint slides of the results of the note analysis and the copy-paste analyses. The 5 focus group sessions were recorded, and transcripts were made for all but one of the focus groups.

The UW Institutional Review Board approved this work and designated it as “minimal risk.”

## Results

From 1000 randomly selected hospital admissions to the Medicine service, we obtained 4938 inpatient progress notes written by 418 authors, an average of 4.9 daily progress notes per patient admission.

**Figure 1:**
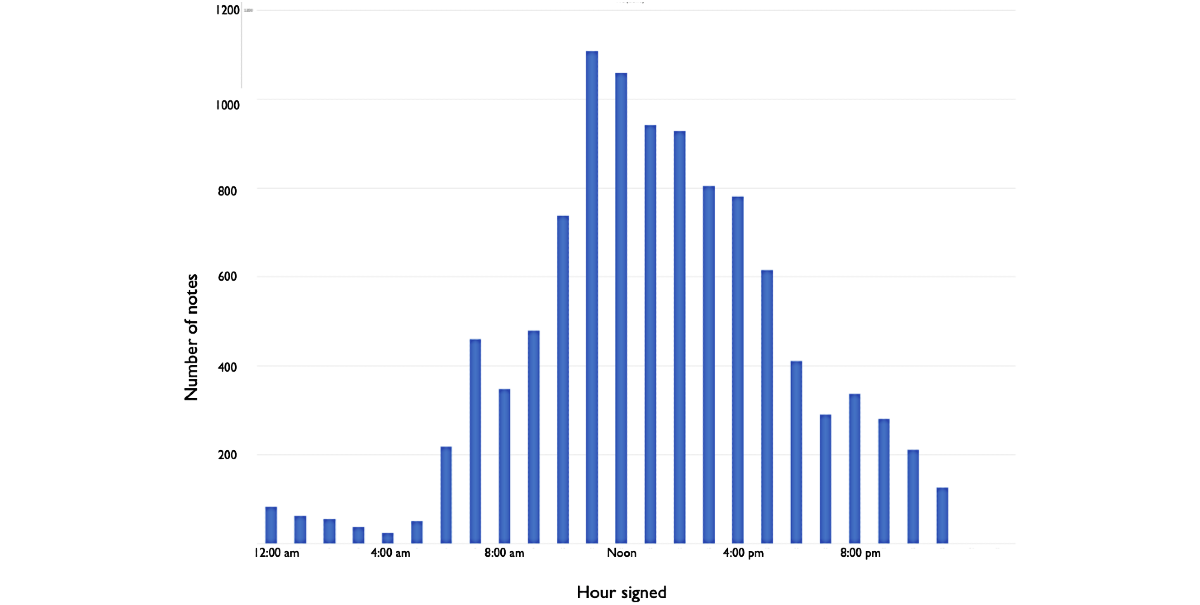
The time at which progress notes were signed by the author.

**Figure 2:**
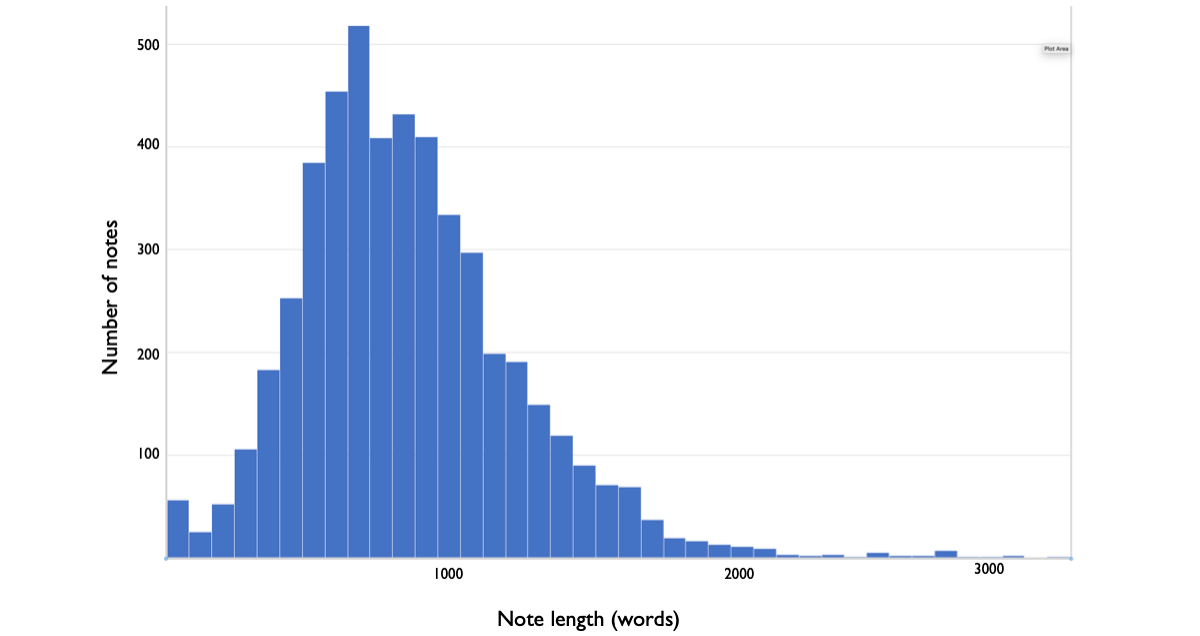
Distribution of note length in words (the programming code used to calculate note length is available from the authors).

**Figure 3:**
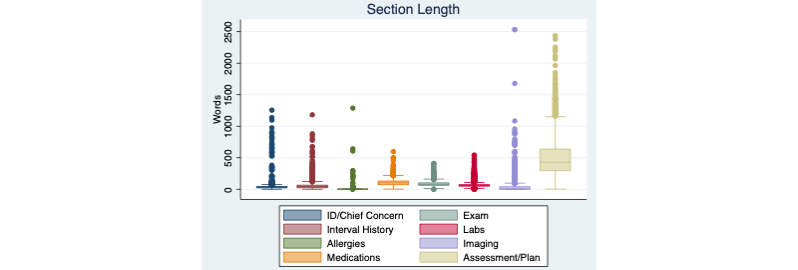
Length of note sections. Box and whisker plots of the number of words in each note section is shown. Box shows IQR. ID: identification.

**Figure 4:**
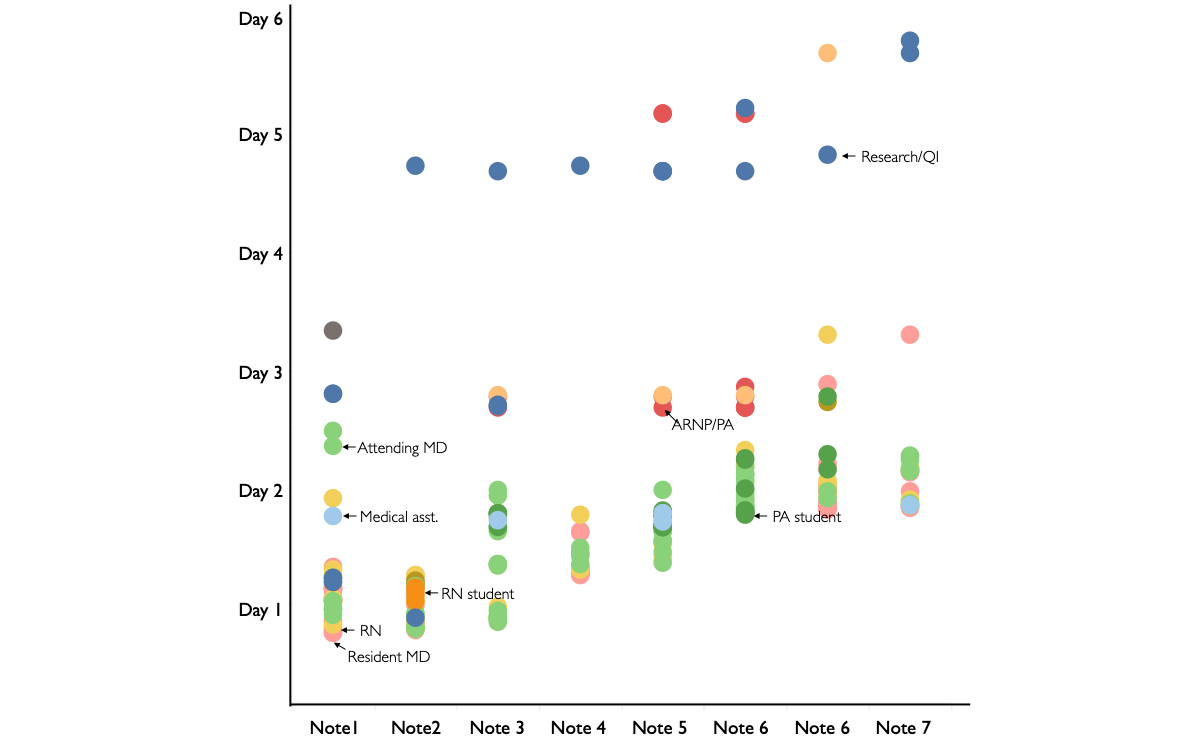
Display of note readership data for 7 notes. Each colored dot indicates who (role) read the note and when over several days. Different colors indicate different roles. Each column of dots shows the readership of one progress note. QI: quality improvement, ARNP: advanced registered nurse practitioner, PA: physician assistant, RN: registered nurse, MD: medical doctor.

The results of the note analysis are summarized in Figures 1-4. The average note length was 886 words (median 827), and most of the length (~500 words) was in the Assessment & Plan section, which comprised on average two-thirds of the note. There was marked variation in note length—some notes contained over 2000 words in the Assessment & Plan section alone. The Interval History and Physical Exam sections were among the shortest sections. These findings reflect the common practice of copying one day’s Assessment & Plan into the next day’s note and appending each day’s assessment to those of the previous days. (While the History and Physical sections are also frequently copied, the lack of appending new information to old information prevents these sections from lengthening over the hospital course.)

Progress note authors signed their notes at various times of the day as shown in Figure 1. A total of 29% (n=1432) of notes were signed after 4 PM, and some were signed as late as 10 PM.

**Figure 5:**
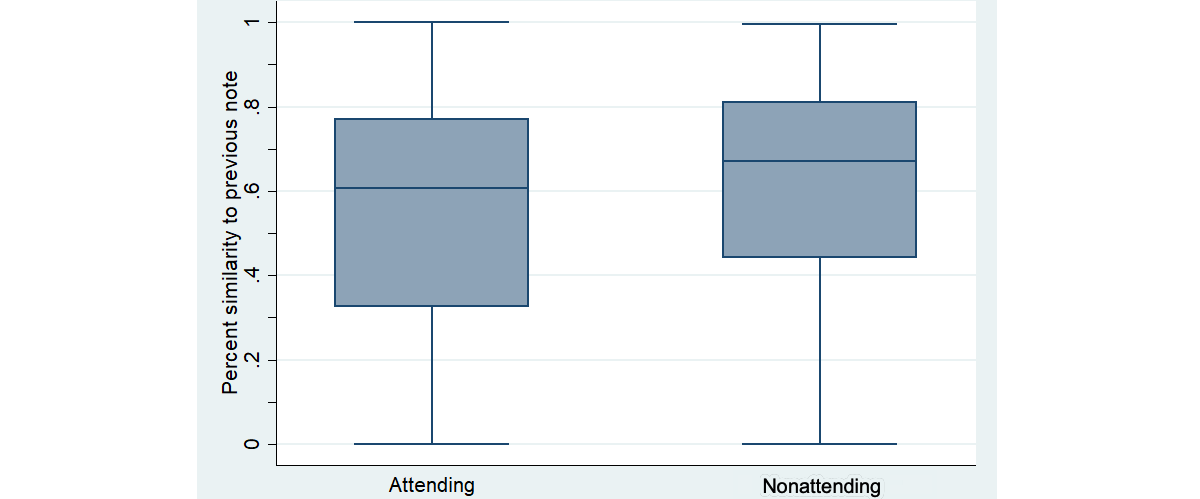
Similarity by author training level. Box and whisker plots of the similarity of notes calculated using natural language processing methods [16,17] are shown. Box shows IQR.

**Figure 6:**
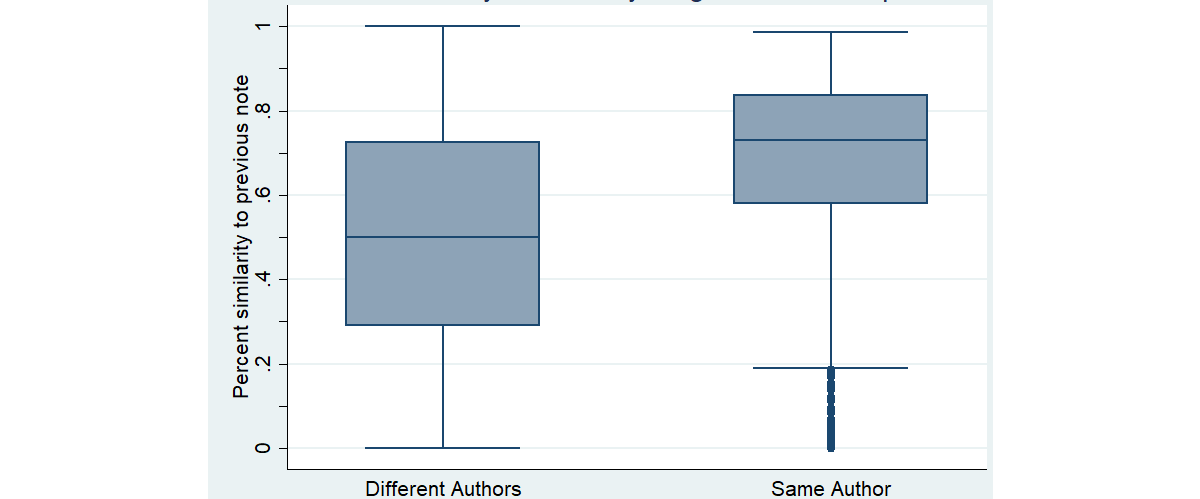
Similarity by original authorship. Box and whisker plots of the similarity of notes calculated using natural language processing methods [16,17] are shown. Box shows IQR.

Notes had high levels of similarity to the prior day’s note on the same patient. The median note similarity was 66% using the methods described above. While note similarity was high for all author types (Figures 5 and 6), it was higher for trainees than attendings (*P*<.001), and higher when both notes were written by the same author rather than by different authors (*P*<.001) (Figure 6).

We conducted manual reviews of note pairs to assess the clinical importance of note similarity. Preserved content from one day to the next was visually highlighted, using the CopyFind program [18]. Physician reviewers then assessed the preserved content for risk using the Hammond scale [18] (Textbox 3). The results showed that 26% (23/88) of the pairs were assessed to have minimal or some risk because of human copying; a second set of reviews found 17% (5/29) of the pairs had minimal or some risk in the preserved content. In this sample then, about 1 in 5 notes had clinical risk associated with preserved content, which was very likely the result of copy-paste practices. An example of copying assessed to be of minimal risk (code 4) was including the phrase “Gen Surg to take to the OR today” when this happened the day before. An example of copying regarded to be of some risk (code 5) was when the History section was completely copied from the day before, incurring a legal risk of fraud. In the Plan section of the same note was the phrase “will obtain MRI” copied forward, but the MRI (magnetic resonance imaging) was obtained the day before as evidenced by the results of the MRI appearing elsewhere in the note.

**Risk scale for duplicated material appearing in notes.**

**Figure 7:**
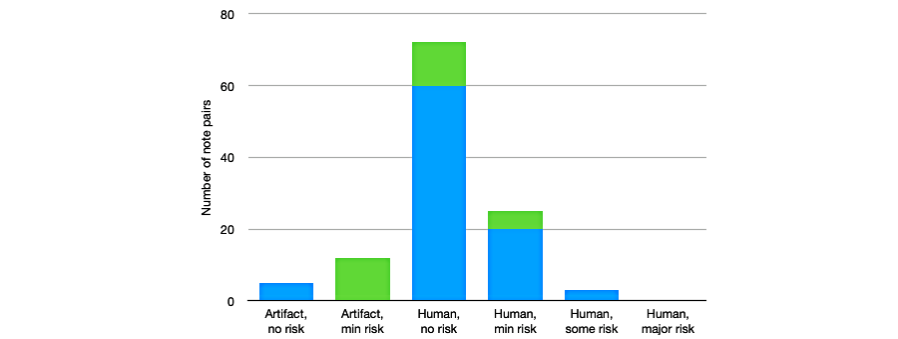
Copy-paste risk, assessed using the Hammond risk scale [18]. Colors represent judgment of risk assigned by the 2 physicians who analyzed a sample of note pairs. One physician assigned scores to 100 note pairs (blue) and one assigned scores to 25 note pairs (green).

The scale below is used to assess the risk associated with duplicated note text, derived from Hammond et al [18] (Figure 7).

Code risk description:

- 1 = Artifact, not misleading, no risk
- 2 = Artifact, minimally misleading, minimal risk
- 3 = Human, not misleading, no risk
- 4 = Human, minimally misleading, minimal risk
- 5 = Human, misleading, some risk
- 6 = Human, clinically misleading, major risk

**Figure 8:**
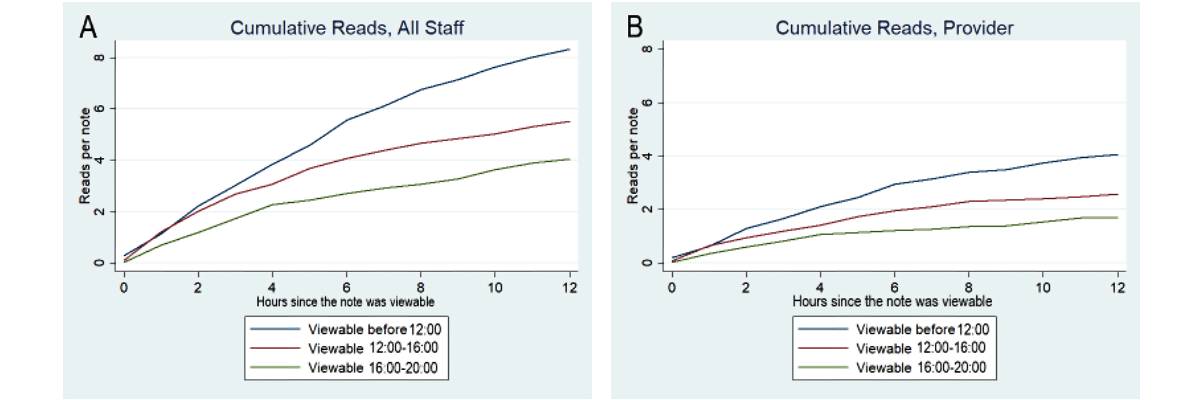
Note reads by published time. Plots show the cumulative number of all staff (A) and providers (B) who read notes as a function of the time since the note was viewable (signed).

Note-reading practices were analyzed on a random subset of 250 notes (limited by auditing time constraints). The 250 notes in this sample were similar to the overall progress note library: the authors who wrote them were representative of the pool of authors in the larger library, and because they were randomly sampled, other note characteristics (eg, time written, length) were similar to the overall note library. This sample had 4036 note views, an average of 16.1 views per note, which includes many types of professionals beyond bedside nurses and physicians. Figures 8A and 8B describe note readership by note sign time. Notes signed before noon were read more than notes signed between noon and 4 PM (*P*=.002), and those signed between noon and 4PM were read more than those between 4 PM and 8 PM (*P*=.05), with successively fewer reads for notes signed between 4 PM and 8 PM. Analysis of note reading by role revealed that more notes were read by providers (physicians, nurse practitioners, physician assistants, medical students) when published before noon (ie, early in the day) (*P*=.003). Nursing readership was not correlated with the time the notes were signed (*P*=.95).

### Focus Groups

#### Note Authors

We learned that note authors vary widely in how they use progress notes (ie, section of note reviewed, such as Labs, Scheduled Medications, etc), and in the purposes for which they use them, such as regarding them to be a billing document, as a “note to self” to remember important items from one day to the next, and as a communication tool to colleagues. Note authors mentioned this as one reason there is so much heterogeneity in the length and content of inpatient progress notes.

#### Note Consumers

Nursing and other ancillary service staff said they value medical progress notes and prefer detailed notes. They regarded them to be an important communication tool that helps them understand what is going on with patients and the plan for the day and for the hospital stay. They frequently used the progress note to assist their communication with families. They reported wanting to see more details regarding the disposition plan and preferred to have the Labs and Radiology sections deleted.

#### Leaders

Leaders believed the collective memory in notes to be important to prevent “reinventing the wheel.” They believed that the primary focus of a note should be communication and patient safety, but that we should also make trainees aware of future billing pressures when they finish training and are able to bill. They felt, as a group, that the medication list is important to include in progress notes.

### Readership

Figure 3 shows that notes are read by authorized users from a wide range of disciplines, including registered nurses, physicians, and administrators.

## Discussion

Our analysis of a large set of randomly selected hospital progress notes found that notes were often long and highly similar to the prior day’s note. Additionally, notes that were signed later were read less frequently; about one-third were signed after 4 PM when their value as a communication tool for other providers is diminished. Given the vast quantity of physician hours spent on their creation, inpatient progress notes have been relatively neglected as an area of study. Limitations in duty hours, increased patient complexity, and decline in physician morale suggest a need to re-evaluate our note-writing practices.

We found broad variation in note-writing and reading practices. Some notes in our sample were over 2000 words (approximately half the length of this paper); this may reflect the common practice of copying one day’s assessment into the next day’s note and adding each day’s findings to those of the previous days. The progress notes often become a running summary of the hospitalization.

Our analysis showed a high degree of similarity between notes on the same patient on successive days. Though methods for measuring note content similarity vary across studies [19], it is interesting that others also noted this [20,21]. Note similarity could occur from either note templating or from copy-paste; we believe that the high similarity in our sample was mainly due to copy-paste, as the most heavily templated sections of our progress note (Allergies, Medications, and Labs) make up just 22% of the average note length. Our focus group with note authors provided further evidence that this was the case.

Note similarity was high across training levels, and while trainee notes had more similarity than attending notes, attending notes were still over 50% similar to the prior day’s note. Perhaps equally concerning was a high degree of similarity between notes with distinct authors. While our Medicine community sees the progress note as more of a living, communal document (where the practice of copy-paste is not viewed as flagrant plagiarism), this practice nevertheless is a clear setup for miscommunication and medical error [22,23]. Indeed, manual review revealed that 1 in 5 notes had clinical risk associated with preserved content, which was very likely the result of copy-paste practices. Reassuringly, this was primarily in the minimal-risk category with no examples of major human risk captured in the small sample we reviewed. This may suggest that careful use of copy-paste with a diligent review (defined as a review sufficient to assure that the note is accurate on the day it is written) of the information that is carried forward is not associated with a major risk of error.

Progress notes in our sample were read frequently by nonprimary team members. Readership of timely clinical data (within 12 hours of note signing) was highly sensitive to the time of day the note was signed, with significantly more notes read if signed before noon. This was especially true of physicians, who tend to have day schedules on the acute care services and are thus unlikely to view a note that is published late in the evening (as many notes are). Nurse views were not sensitive to note-signing time, likely reflecting the presence of night-shift nurses, who also want to use the progress note as a means to communicate the plan of care. (However, if a night-shift nurse reads a note, the content from the morning rounds is over 12 hours old.)

In the focus groups, we heard many and different reasons nurses and physicians value progress notes. Some nurses read them soon after they were created even if it was late in the day and appreciated the detail of Medicine progress notes compared with other services. For nurses, these notes served as an important communication tool to help them understand the Medicine team’s plan. Most expressed interest in more information tailored to their needs. Note authors—largely house staff and hospitalists—also valued notes, though what they valued differed by group. House staff are charged with tracking details, critical and minor, and used their notes for their “future selves”—as a way to remind them of what is to be done and for the collective memory of what has occurred. For hospitalists, there was wide variation in the attitude toward progress note writing, with some hospitalists favoring a more succinct note that concisely summarizes why the patient remains hospitalized and focuses on changes in patient status and plan for that day, while others (especially those who also worked nights) also focused on the importance of a note as a communication tool. Ultimately, the use of the note as a repository or collective memory of hospital course was a hotly contested topic among the hospitalists depending on their attitude toward and group practice with regards to interim summary writing. This suggests a need for direction from leadership regarding the use and role of medical inpatient progress notes versus interim summaries as collective memory of a patient’s hospital course.

Recent literature offers additional perspectives and hopes for progress notes. We know that attendings and house staff physicians differ in their perception of note quality but agree that communication is an important purpose for them [24]. Some senior authors advocated restoring the story to clinical notes [25,26]. If this is an objective, using voice to create notes could fit this into the workflow because it may be faster than typing a more narrative note. Creating a wiki-style note, broadly adopting the APSO (Assessment, Plan, Subjective, Objective) format, utilizing vendor tools to create an ongoing hospital course, or drastically shortening notes were not explored in this study but may be embraced by a segment of physicians.

These results have implications for those who develop electronic documentation systems used in EHRs. Documentation methods that facilitate the completion of notes sooner in the day may result in greater readership, fulfilling an important communication objective for electronic notes. However, the opportunity cost of earlier documentation may be diverting physician hours away from other time-sensitive tasks, such as contacting specialists or meeting with families. Permitting the simple creation of a summary of the hospital course, outside of the progress note itself, could result in more succinct notes that convey what progress occurred that day, and would be simpler to read. Developing note-writing tools that permit more rapid note creation, tailored to the aptitudes of the note author, such as use of voice [27,28] or scribes, could permit the notes to be signed earlier in the day, resulting in increased readership, which is an important objective of the daily progress note.

Templates can also speed note writing but is done so at the risk of retaining content of the history or physical exam, which is included in some templates. Many clinicians who read notes are most interested in what the note author has to say rather than what is within the template text. Every change to note-writing approaches has potential for side-effects. For example, methods that reduce the time to create notes and that permit them to be completed earlier in the day may also decrease note quality.

There is an important role for hospital leaders in setting the direction for this critical part of clinical care and training [29]. As a result of this work, our community has expressed agreement on increasing the communication value of progress notes, in part by making it possible for them to be completed earlier, and to reduce the time devoted to writing them.

## Abbreviations

APSO: Assessment, Plan, Subjective, Objective
EHR: electronic health record
MRI: magnetic resonance imaging
UW: University of Washington
